# Rapid and strain-specific resistance evolution of *Staphylococcus aureus* against inhibitory molecules secreted by *Pseudomonas aeruginosa*

**DOI:** 10.1128/mbio.03153-22

**Published:** 2023-08-30

**Authors:** Selina Niggli, Lukas Schwyter, Lucy Poveda, Jonas Grossmann, Rolf Kümmerli

**Affiliations:** 1 Department of Quantitative Biomedicine, University of Zurich, Winterthurerstrasse, Zurich, Switzerland; 2 Functional Genomics Center Zurich, ETH Zurich and University of Zurich, Winterthurerstrasse, Zurich, Switzerland; 3 Swiss Institute of Bioinformatics (SIB) Quartier Sorge-Batiment Amphipole, Lausanne, Switzerland; University of Pittsburgh, Pittburgh, Pennsylvania, USA; The University of Iowa, Iowa City, Iowa, USA

**Keywords:** microbe-microbe interactions, polymicrobial infections, pathogen evolution, competition, resistance evolution, nosocomial pathogen

## Abstract

*Pseudomonas aeruginosa* and *Staphylococcus aureus* frequently occur together in polymicrobial infections, and there is evidence that their interactions negatively affect disease outcome in patients. At the molecular level, interactions between the two bacterial species are well-described, with *P. aeruginosa* usually being the dominant species suppressing *S. aureus* through a variety of inhibitory molecules. However, in chronic infections the two species interact over prolonged periods of time, and *S. aureus* might be able to evolve resistance against inhibitory molecules deployed by *P. aeruginosa*. Here, we used experimental evolution to test this hypothesis by exposing three different *S. aureus* strains (Cowan I, 6850, and JE2) to the growth-inhibitory supernatant of *P. aeruginosa* PAO1 over 30 days. Prior to evolution, we found that *S. aureus* strains were inhibited by secreted compounds regulatorily controlled by the *Pseudomonas* quinolone signal quorum-sensing system. Following evolution, inhibitory effects were significantly attenuated, and we observed that adaptations were *S. aureus* strain specific and involved the upregulation of virulence traits such as staphyloxanthin production and the formation of small colony variants. At the genetic level, mutations in membrane transporters (known to be involved in antibacterial uptake) were the most frequent evolutionary targets. Our work indicates that adaptations of *S. aureus* to *P. aeruginosa* occurs rapidly and affect both virulence trait expression and membrane transporter functionality. Thus, pathogen evolution could promote species co-existence and complicate treatment options in infections.

**IMPORTANCE:**

Polymicrobial infections are common. In chronic infections, the different pathogens may repeatedly interact, which could spur evolutionary dynamics with pathogens adapting to one another. Here, we explore the potential of *Staphylococcus aureus* to adapt to its competitor *Pseudomonas aeruginosa*. These two pathogens frequently co-occur, and *P. aeruginosa* is seen as the dominant species being able to displace *S. aureus*. We studied three different *S. aureus* strains and found that all became quickly resistant to inhibitory compounds secreted by *P. aeruginosa*. Our experimental evolution revealed strains-specific adaptations with three main factors contributing to resistance evolution: (i) overproduction of staphyloxanthin, a molecule protecting from oxidative stress; (ii) the formation of small colony variants also protecting from oxidative stress; and (iii) alterations of membrane transporters possibly reducing toxin uptake. Our results show that species interactions can change over time potentially favoring species co-existence, which in turn could affect disease progression and treatment options.

## INTRODUCTION

Bacterial infections are frequently caused by multiple species (1 – 3) and such polymicrobial infections can be more severe than the respective mono-species infections (4 – 7). Because of the detrimental effects for patients, there is high interest in understanding the mechanisms of bacterial interactions and how they affect ecological dynamics between co-infecting species. Interactions between the two opportunistic human pathogens *Pseudomonas aeruginosa* (PA) and *Staphylococcus aureus* (SA) have attracted particularly high attention (8, 9). This is because they are major pathogens and often co-occur in the lungs of cystic fibrosis (CF) patients and in burn and chronic wound infections (10 – 13). Co-infections with PA and SA can be associated with more severe disease outcomes in humans (13, 14), and studies in animal models confirmed increased virulence and compromised antibiotic treatment options during co-infections (15 – 17).

Laboratory studies established a solid mechanistic understanding of how the two species interact. While positive relationships were observed in the context of metabolic interactions (18) and protection from antibiotics in biofilms (19), antagonistic interactions often prevail between the two species (20 – 22). Overall, PA seems to dominate the interactions through the production and secretion of a variety of inhibitory molecules, such as the staphylolytic protease LasA, siderophores, and the respiratory chain inhibitors 2-n-heptyl-4-hydroxyquinoline N-oxide (HQNO) and phenazines (23 – 28). There is also increasing knowledge on ecological factors that influence interactions patterns. For example, during early childhood, CF lungs are first colonized by SA followed by PA later on, with SA abundance tending to decrease when PA increases (29). In the context of wound infections, we also have an increased understanding about the spatial localization of the two species, where it seems that PA and SA occupy different niches (30, 31).

In contrast to these mechanistic and ecological insights, we know little about whether interactions between PA and SA can evolve (32). Species co-evolution is likely to occur in chronic infections, where pathogens repeatedly interact, and is predicted to alter disease parameters and treatment strategies. In the context of interactions between PA and SA, we hypothesize that SA could adapt and become resistant to PA inhibitory molecules. Such adaptation could, in turn, foster co-existence of the two species, and potentially contribute to the clinically observed higher virulence, increased morbidity, and treatment complications (13, 14).

Here, we use a combination of experimental evolution, phenotypic screening, and whole-genome sequencing of evolved clones to test our hypothesis regarding resistance evolution. We first conducted a series of experiments with three different SA strains to show that all of them are inhibited by secreted compounds present in the supernatant and regulated by the *Pseudomonas* quinolone signal (PQS) quorum-sensing system of the PA strain PAO1. We then exposed the three SA strains to either the PA supernatant (containing the inhibitory molecules, mixed with fresh medium) or a control medium for 30 consecutive days, by transferring evolving cultures every 48 hours to fresh conditions. Following experimental evolution, we screened evolved SA clones from replicated populations for resistance phenotypes and explored whether virulence traits were under selection. Finally, we sequenced the whole genome of 150 clones to determine the genetic basis of SA resistance evolution against inhibitory molecules from PA.

## RESULTS

### Growth of SA strains is reduced in the presence of PA supernatant

**Fig 1:**
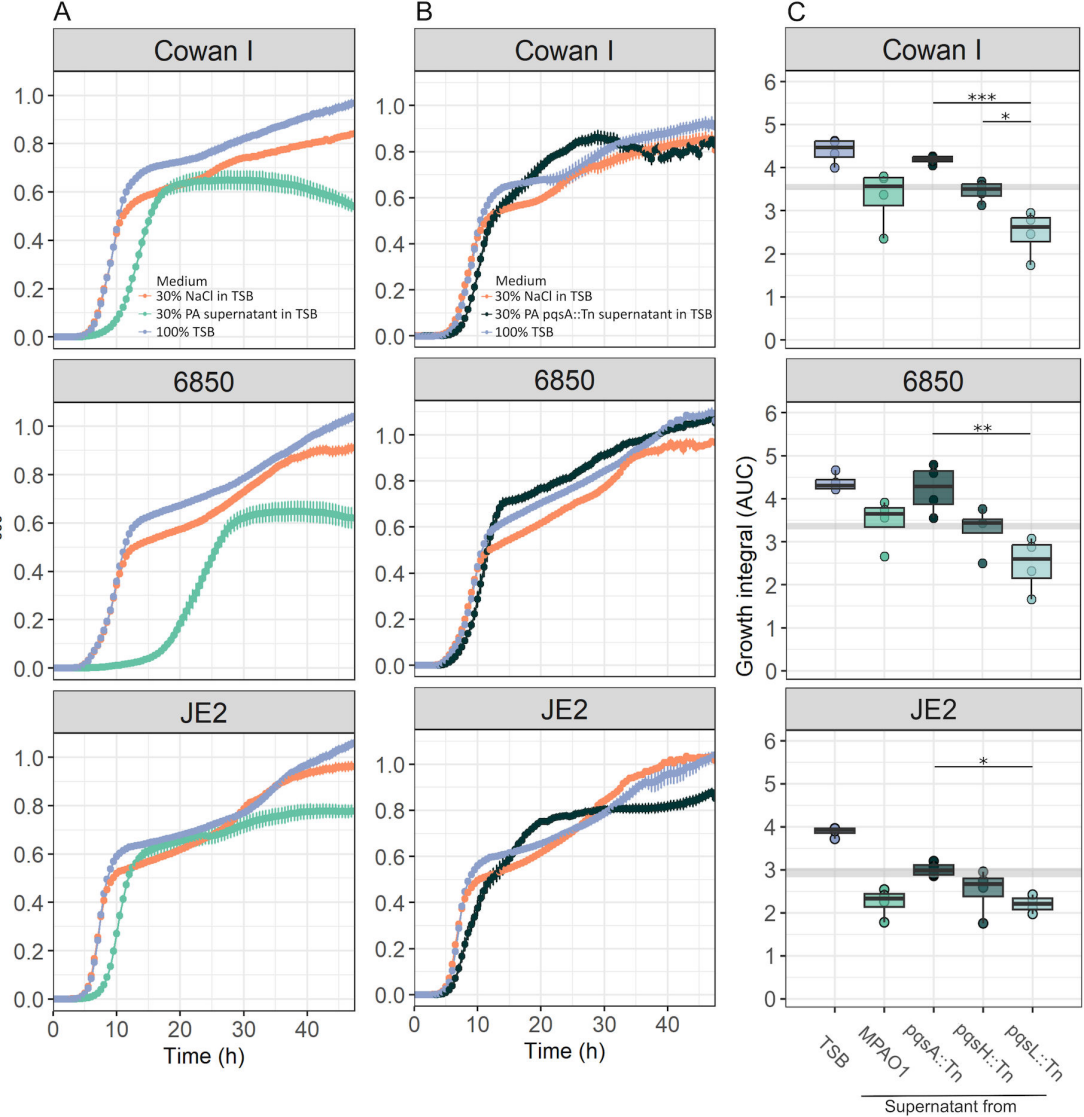
Effect of supernatants from PA wild type and various PQS mutants on three SA strains, Cowan I, 6850, and JE2. (**A**) The supernatant of the PA wild type significantly reduced the growth (particularly extended the lag phase) of all three SA strains. SA strain growth was followed over 48 hours at 37°C under three different conditions: 30% NaCl in tryptic soy broth (TSB) (control 1), 30% PA supernatant in TSB (experimental treatment), and 100% TSB (control 2). Data are from seven independent experiments each featuring four replicates per strain and condition. Shown is mean growth ± standard error. Statistical comparisons of growth parameters are shown in Fig. S1. (**B**) The inhibitory effect on SA strains is reduced when exposed to the supernatant of MPAO1*ΔpqsA*. The lag phase is still slightly extended, but the yield is no longer affected. Data are from two independent experiments with eight replicates per strain and condition. (**C**) Comparison of the growth integral [area under the curve (AUC)] of SA strains exposed the supernatants of four different PA strains: MPAO1 wild type, MPAO1*pqsA::Tn* (Tn-pqsA, deficient to produce all PQS-regulated traits), MPAO1*pqsH::Tn* (Tn-pqsH, producing HHQ and HQNO), and MPAO1*pqsL::Tn* (Tn-pqsL, producing HHQ and PQS). The three mutants and the corresponding wild type are from the Manoil transposon mutant library (33). For this analysis, we considered the first 15 hours of growth during which inhibitory effects were most prominent. Data are from one experiment with four replicates per strain and condition. Asterisks denote significant differences: ***P < 0.001; **P < 0.01; *P < 0.05; OD_600_, optical density at 600 nm.

Previous work showed that PA can inhibit SA via a diverse set of secreted molecules, including proteases, biosurfactants, siderophores, and toxic compounds (8). To test whether our three SA strains (Cowan I, 6850 and JE2, Table S1) are also inhibited by PA, we exposed them to the sterile-filtered supernatant of PA, harvested from overnight cultures [30% supernatant in 70% fresh tryptic soy broth (TSB)]. We found that all three SA strains were negatively affected by the PA supernatant compared to growth under control condition (30% NaCl in 70% TSB, Fig. 1A). Exposure to PA supernatant significantly reduced overall growth performance [Fig. S1A, analysis of variance (ANOVA) on growth integrals followed by Tukey’s honestly significant difference (HSD) pairwise comparisons: p_adj_ < 0.0001 for all SA strains], and specifically extended the lag phase of all SA strains (Fig. S1B). We also observed SA strain-specific responses (Fig. 1). Cowan I featured an intermediate extension of the lag phase, a premature stationary phase, followed by a decrease in optical density at 600 nm (OD_600_) possibly indicating cell death. 6850 suffered from an extremely long lag phase extension and also showed a premature growth stop but no OD_600_ decline. JE2 was least affected by the PA supernatant. Its lag phase extension was not as pronounced, and it grew steadily afterwards.

We also conducted the reciprocal experiments by exposing PA to the supernatants of the three SA strains but found no inhibition (Fig. S2). Taken together, our supernatant assays reveal unilateral inhibition: PA produces inhibitory molecules that negatively affect the growth of genetically different SA strains.

### The PQS pathway is involved in SA growth inhibition

To identify possible PA pathways or traits involved in the inhibition of SA, we screened a small panel of seven PA mutants and checked whether the inhibitory effects of their supernatants toward SA were altered. We found that supernatants of six mutants still inhibited all SA strains (Table S2) and two of them (PAO1*ΔpvdDpchEF* and PAO1*ΔlasR*) inhibited SA strains even more (particularly 6850) than the PA wild-type supernatant (Table S2). While we do not know the nature of this increased inhibition, the results show that siderophores and the staphylolytic protease LasA (controlled by LasR) are not involved in SA inhibition in our experiments. Conversely, we found that the supernatant of PAO1*pqsA::Tn* showed a significantly reduced inhibitory effect on SA (Fig. 1B; Table S2). A closer inspection of the growth curves reveals that the supernatant of PAO1*pqsA::Tn* still reduced the lag phase to some extent but resulted in higher yield at later stages of the growth cycle (Fig. 1B).

PqsA is an anthranilate-coenzyme A ligase that plays a role in PA cell-to-cell communication (quorum sensing) (34). It is directly involved in the synthesis of a family of secondary metabolites, including 4-hydroxy-2-heptylquinoline (HHQ), HQNO, and the PQS. HHQ, HQNO, and PQS have previously been suggested to inhibit a variety of bacterial and fungal species (26, 35 – 37). To explore the role of these three molecules in more detail, we repeated the above assay with supernatants of MPAO1*pqsA::Tn* (producing none of the three molecules), MPAO1*pqsH::Tn* (producing HHQ and HQNO), and MPAO1*pqsL::Tn* (producing HHQ and overproducing PQS). For all three SA strains, we observed a staged effect of these supernatants on SA growth (Fig. 1C). While MPAO1*pqsA::Tn* supernatant had weak inhibitory effects on SA growth, the inhibitory effects increased for MPAO1*pqsH::Tn* and were strongest for MPAO1*pqsL::Tn* supernatants. As before, we observed strong effects on the lag phase of SA strains, while inhibition weakened later during the growth phase (Fig. S3). These results confirm that both HQNO and PQS can inhibit the SA strains, with PQS showing a somewhat stronger effect. Moreover, the results indicate that the inhibitory molecules degrade over time in our assay, allowing SA strains to recover (Fig. 1; Fig. S3). Exposing the three SA strains to synthetic PQS confirmed the inhibitory effect and revealed a dose-dependent reduction in growth (Fig. S4).

### All SA strains evolve resistance to PA supernatant inhibition

We then conducted an experimental evolution experiment to explore whether SA can adapt to the inhibitory compounds secreted by PA (Fig. S5). We can expect resistance evolution because the observed growth reduction (induced either by cell death or longer cell division times) is considerable and should exert a relatively strong selection pressure. We exposed the three SA strains (Cowan I, 6850, and JE2) to either 30% PA supernatant + 70% TSB or to 100% TSB over 30 days. For each strain-condition combination, we had seven replicated populations evolving independently. Overall, there were 42 evolving populations, which we transferred every 48 hours to fresh medium (15 transfers in total).

**Fig 2:**
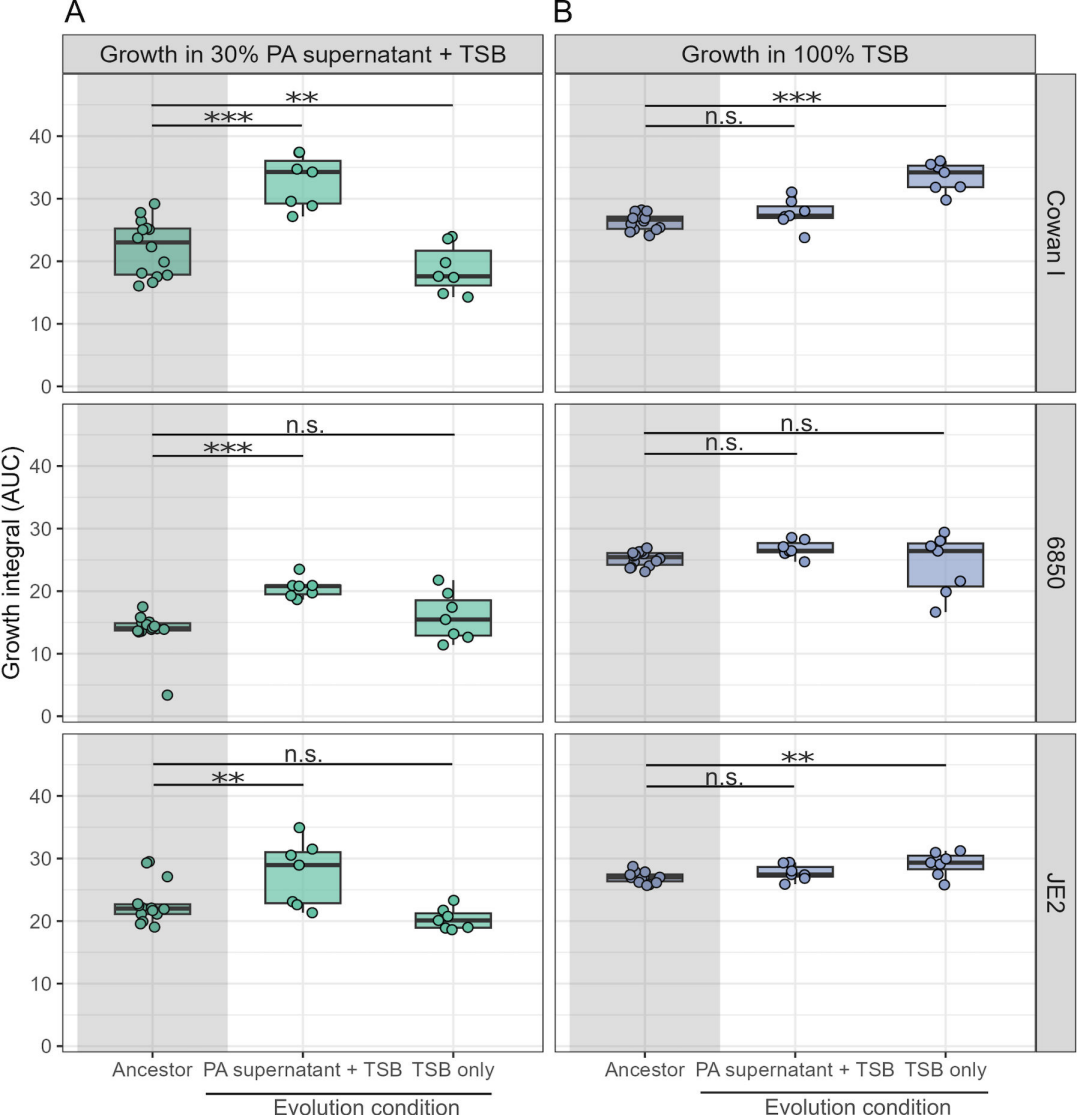
The three SA strains (Cowan I, 6850, and JE2) evolved resistance to inhibitory molecules produced by PA when exposed to its supernatant during experimental evolution. Populations of SA strains (*n* = 7 per strain) either evolved in 30% PA supernatant + 70% tryptic soy broth (TSB) or 100% TSB over 30 days with transfer to fresh medium every 48 hours. (**A**) Growth of ancestral (gray-shaded area) and evolved populations in medium containing 30% PA supernatant + 70% TSB. SA populations evolved in the presence of PA supernatant significantly improved growth, while populations evolved in TSB alone are still inhibited by the supernatant. (**B**) Growth of ancestral (gray-shaded area) and evolved populations in 100% TSB medium. SA populations evolved in the presence of PA supernatant did not improve growth in TSB alone, while populations evolved in TSB did so for two of the three SA strains. Box plots show the median (bold line), the first and third quartiles, and the 1.5* inter-quartile range (IQR, whiskers) or the range from the lowest to highest value if all values fall within the 1.5* IQR. Dots represent the individual populations (mean across four technical replicates). Asterisks denote significant differences between the ancestral and evolved populations. ***P < 0.001; **P < 0.01; AUC, area under the curve; n.s., not significant. *P*-values are adjusted by the false discovery rate method.

Following experimental evolution, we found that populations that had evolved in the presence of PA supernatant showed significantly improved growth performance compared to their respective ancestors when exposed to the PA supernatant, indicating the evolution of resistance [Fig. 2A, *t*-tests (two-tailed), Cowan I: t_12_ = 11.41, *P* < 0.0001; 6850: t_12_ = 6.31, *P* = 0.0002; JE2: t_12_ = 3.61, *P* = 0.0072]. Conversely, all populations that evolved in 100% TSB were still fully or even more susceptible to the PA supernatant compared to the ancestor (Fig. 2A, Cowan I: t_12_ = −3.98, *P* = 0.0055; 6850: t_12_ = 1.00, *P* = 0.4039; JE2: t_12_ = −0.87, *P* = 0.4368). Next, we tested whether evolved populations showed TSB-specific adaptations. Indeed, we found that populations of Cowan I and JE2 that had evolved in 100% TSB significantly increased their growth in this medium but not in 6850 (Fig. 2B, Cowan I: t_12_ = 11.09, *P* < 0.0001; 6850: t_12_ = −0.77, *P* = 0.4590; JE2: t_12_ = 3.70, *P* = 0.0072). In contrast, populations of all three SA strains that had evolved in the presence of PA supernatant did not improve growth in TSB (Fig. 2B, Cowan I: t_12_ = 2.32, *P* = 0.0663; 6850: t_12_ = 1.41, *P* = 0.2467; JE2: t_12_ = 1.94, *P* = 0.1137). These results show that all three SA strains have specifically adapted to the presence of PA supernatant and not to TSB, and that these adaptations reduce the growth inhibition originally imposed by the PA supernatant.

We then isolated five random clones per independently evolved population (210 clones in total) and repeated the above growth assays with the evolved clones. The results confirmed our population-level analysis of resistance evolution but also revealed considerable variation among clones in their growth (Fig. S6). For example, several clones of Cowan I and JE2 showed highly improved growth in PA supernatants, while others were still inhibited similar to the ancestral wild type. This variation suggests that populations are heterogeneous and consist of multiple different genotypes.

### Clones evolved in PA supernatant show strain-specific resistance to PQS

**Fig 3:**
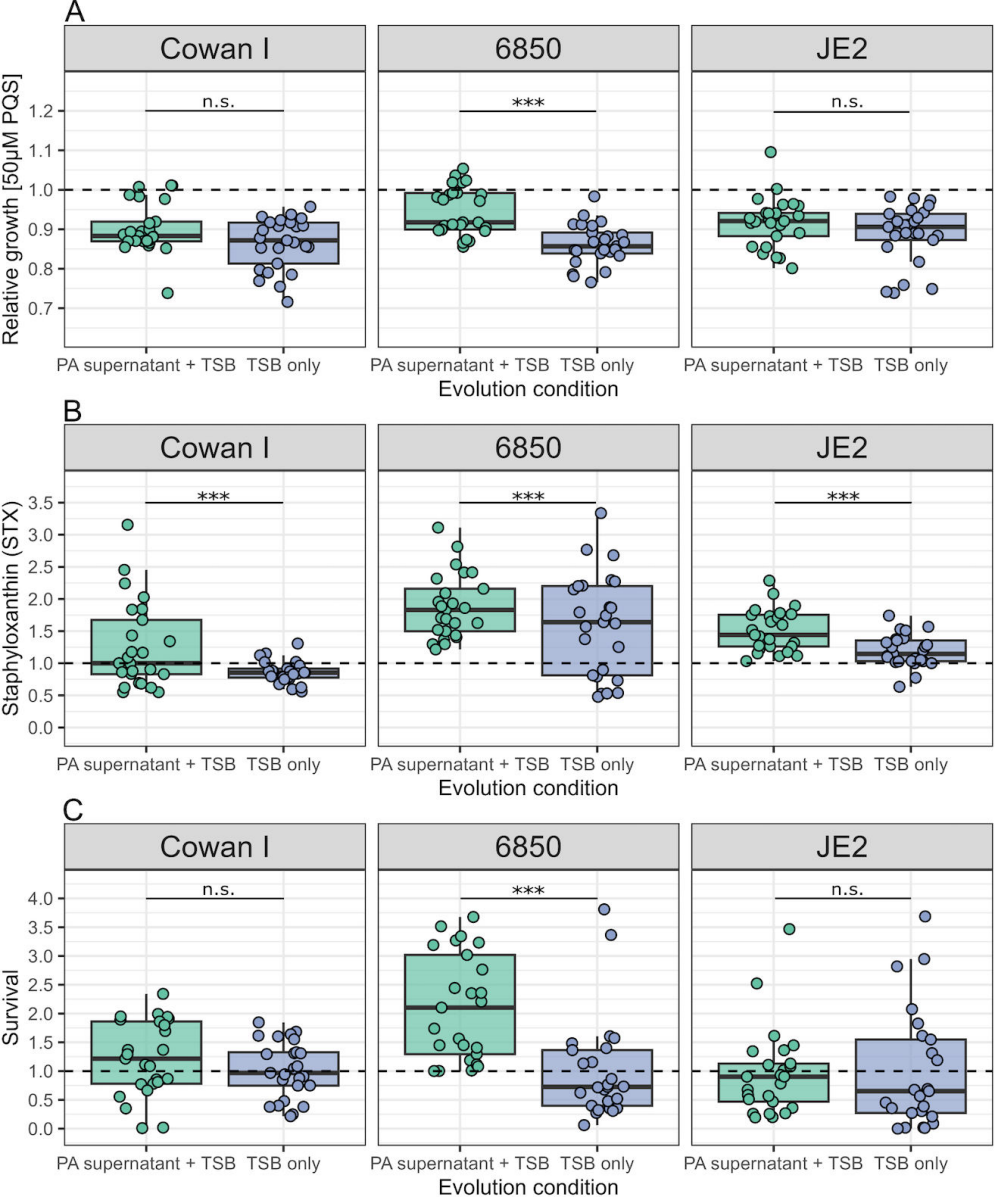
Change in growth under *Pseudomonas* quinolone signal (PQS) exposure, staphyloxanthin (STX) production, and survival in the presence of hydrogen peroxide (H_2_O_2_) after experimental evolution varies across SA strains and media conditions. (**A**) Growth of evolved clones of Cowan I, 6850, and JE2 when exposed to 50 µM PQS expressed relative to the untreated respective ancestor (dashed line). (**B**) Production of STX for evolved clones of Cowan I, 6850, and JE2 expressed relative to the respective ancestor (dashed line). (**C**) Survival of evolved clones of Cowan I, 6850, and JE2 after exposure for 1 hour to 1.5% H_2_O_2_, with the survival data being log(x + 1)-transformed and expressed relative to the respective ancestor (dashed line). Every dot represents an evolved clone (*n* = 25 for each combination of SA strain and evolution condition) and shows the average value from two independent experiments. Box plots show the median (bold line), the first and third quartiles, and the 1.5* inter-quartile range (IQR, whiskers) or the range from the lowest to highest value if all values fall within the 1.5* IQR. Asterisks denote significant differences between the two evolution conditions (Tukey’s HSD with adjusted *P*-values). ***P < 0.001; n.s., not significant; TSB, tryptic soy broth.

We then asked whether the increased growth performance of SA clones evolved in PA supernatant is linked to PQS resistance. Hence, we exposed evolved clones to 50 µM PQS, representing the intermediate inhibitory concentration for the ancestral strains (Fig. S4). Note that we restricted all phenotypic screens to 150 clones from 30 out of the 42 populations, as this sample size matched our genome sequencing contingent. We found that the relative growth of evolved SA clones under PQS exposure was explained by a significant statistical interaction between strain background and the medium the strains evolved in (ANOVA: F_2,144_ = 4.86, *P* = 0.0091, Fig. 3A). Specifically, 6850 clones evolved in PA supernatant grew significantly better under PQS exposure than clones evolved in TSB alone (Tukey’s HSD, p_adj_ < 0.0001) with many clones showing full growth recovery. In contrast, no overall increase in growth was observed for Cowan I (p_adj_ = 0.2519) and JE2 (p_adj_ = 0.5475) clones evolved in PA supernatant under PQS exposure, although a few individual Cowan I clones showed signs of growth recovery.

### Clones evolved in the presence of PA supernatant overproduce staphyloxanthin

PQS and HQNO promote the formation of reactive oxygen species and therefore cause oxidative stress in target cells (38 – 41). Since staphyloxanthin (STX), the golden carotenoid pigment, acts as an antioxidant in SA (42), we tested whether the production of this pigment has changed over evolutionary time in the presence of PA supernatant (43). Indeed, we found that STX production was significantly higher in clones evolved in PA supernatant than in clones evolved in TSB alone (ANOVA: F_1,146_ = 16.37, *P* < 0.0001, Fig. 3B). Moreover, there were significant differences between SA strains (F_2,146_ = 22.93, *P* < 0.0001), with the strongest average increase in STX production occurring in 6850. By contrast, the average increase in STX production was moderate among Cowan I and JE2 clones. In TSB alone, several evolved 6850 clones also showed increased STX production relative to the ancestor. These analyses suggest that STX production is generally under selection in TSB but reaches particularly high levels in the presence of PA supernatant.

### Survival under oxidative stress correlates with PQS resistance and STX production

The above data suggest that SA can become resistant to oxidative stress induced by PQS and HQNO and that upregulation of the antioxidant STX might be involved in this process. To test this hypothesis more directly, we exposed all evolved clones for 1 hour to a 1.5% hydrogen peroxide (H_2_O_2_) solution and explored whether clones evolved in PA supernatant show increased survival under oxidative stress. We observed that the survival of evolved SA clones under H_2_O_2_ exposure was explained by a significant statistical interaction between strain background and the medium the strains evolved in (ANOVA: F_2,144_ = 6.81, *P* = 0.0015, Fig. 3C). Specifically, 6850 clones evolved in PA supernatant had significantly higher survival than clones evolved in TSB alone (Tukey’s HSD, p_adj_ < 0.0001), which was not the case for Cowan I (p_adj_ = 0.949) and JE2 (p_adj_ = 1.0) clones evolved in PA supernatant. When comparing across strains, we found that survival in the presence of H_2_O_2_ correlated positively with growth under PQS exposure for clones evolved in PA supernatant (Pearson’s product-moment correlation, r_73_ = 0.30, *P* = 0.0092, Fig. S7A) and STX production (r_73_ = 0.27, *P* = 0.0207, Fig. S7B). In contrast, such correlations were either negative (survival vs growth under PQS: r_73_ = −0.37, *P* = 0.0010, Fig. S7A) or absent (r_73_ = −0.02, *P* = 0.8920, Fig. S7B) for clones evolved in TSB alone. In sum, our analyses reveal that coping with oxidative stress is a major component of SA adaptation to inhibitory PA supernatant and that the evolutionary response is strongest among clones of 6850.

### SA strains follow divergent and medium-specific evolutionary trajectories

**Fig 4:**
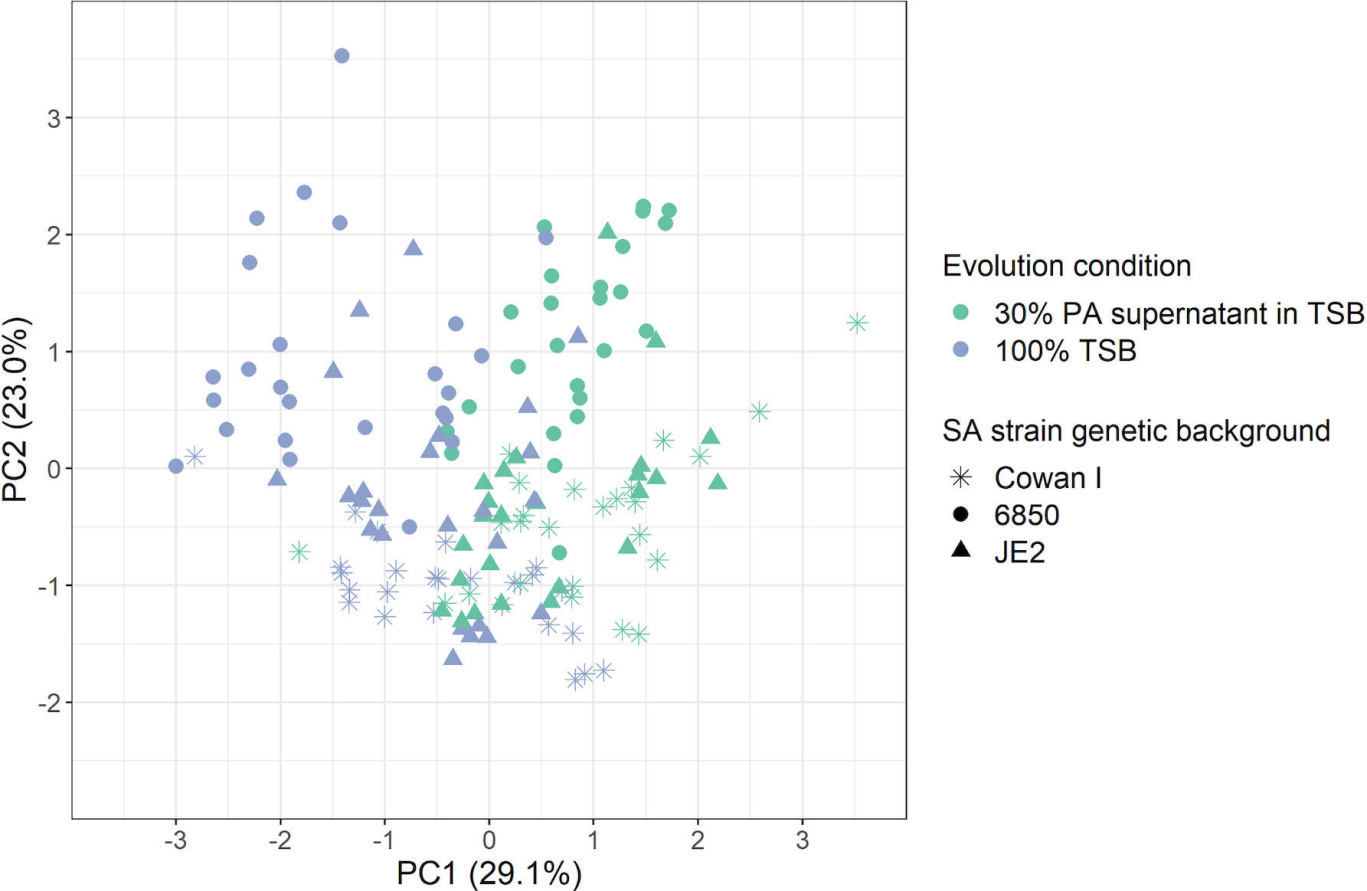
PCA on five phenotypes of 150 evolved clones reveals strain and condition-specific evolutionary divergence. The following five phenotypes were integrated into the PCA: growth in 30% PA supernatant + 70% tryptic soy broth (TSB), growth in 100% TSB, growth under PQS exposure, staphyloxanthin production, and survival in the presence of oxidative stress. The first two principal components (PC1 and PC2) explain 52.1% of the total variance in the data set. Statistical analyses reveal significant divergences in the evolved phenotypes between the three SA strains (Cowan, 6850, and JE2) and the condition they evolved in (30% PA supernatant + 70% TSB, 100% TSB).

To confirm that the observed phenotypic changes during experimental evolution are strain and condition specific, we performed a principal component analysis (PCA) with the following five phenotypes of the 150 evolved clones: (i) growth in 100% TSB; (ii) growth in 30% PA supernatant + 70% TSB; (iii) relative growth under PQS exposure; (iv) STX production; and (v) survival in the presence of H_2_O_2_. We found that evolved clones significantly clustered based on the evolution condition [TSB only vs PA supernatant + TSB: permutational multivariate analysis of variance (PERMANOVA); F_1,144_ = 33.24, *P* = 0.0010, Fig. 4], strain background (F_2,144_ = 18.17, *P* = 0.0010) and their interaction (F_2,144_ = 9.37, *P* = 0.0010).

### Formation of small colony variants and hemolysis maintenance

**Fig 5:**
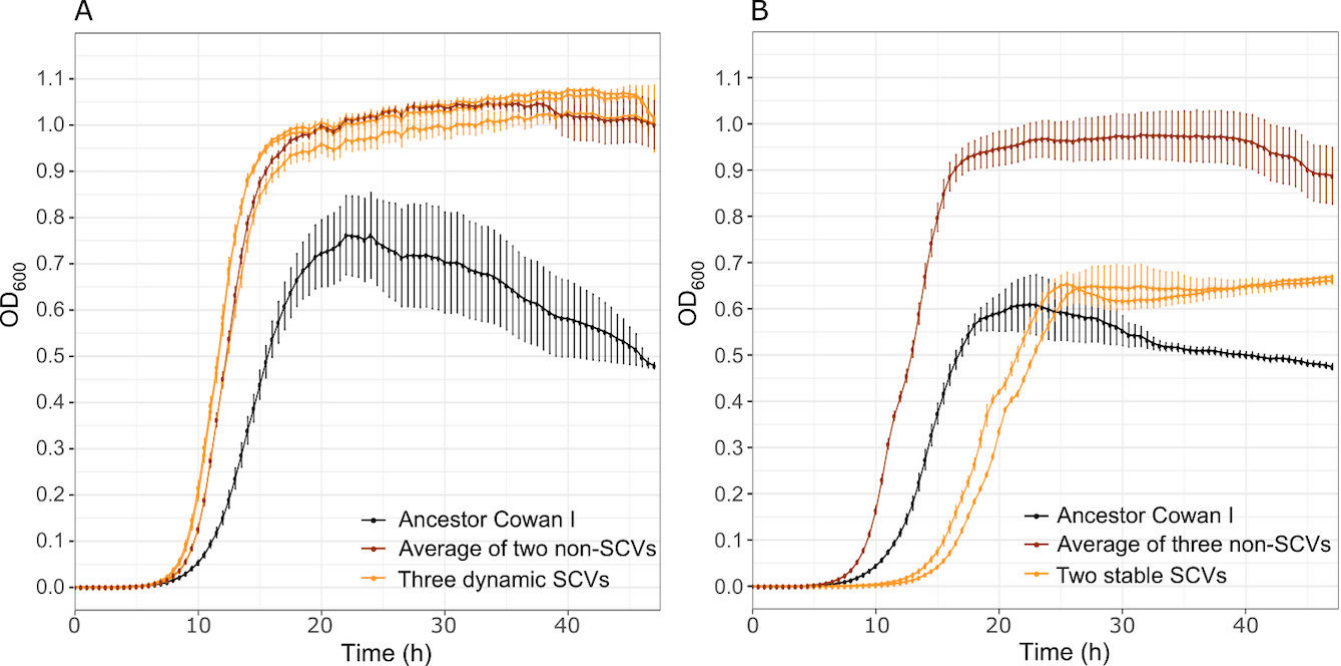
Characteristics of the five small colony variants (SCVs) evolved in the Cowan I background. (**A**) Growth trajectories of the three dynamic/unstable SCVs in 30% PA supernatant + 70% TSB. Compared to the Cowan I ancestor, dynamic/unstable SCVs have a shorter lag phase and a higher yield, which is similar to the normal-sized clones isolated from the same population. (**B**) Growth trajectories of stable SCVs in 30% PA supernatant + 70% TSB. Compared to normal-sized clones from the same population, the two stable SCVs have a longer lag phase and a lower growth yield, but they no longer show the decrease in optical density at 600 nm (OD_600_) that is characteristic for the Cowan I ancestor (see Fig. 1). Curves show the average growth (± standard error) across four independent replicates.

We explored two additional phenotypes that have previously been associated with SA adaptation in chronic infections—small colony variant (SCV) formation and hemolysis—and test whether they also change in response to PA supernatant exposure. SCV formation can offer protection from stressful conditions, including reactive oxygen species (26, 44 – 46). Our population-level screen revealed SCVs in five out of seven Cowan I populations that had evolved in the presence of PA supernatant (Fig. S8). SCVs also surfaced in populations of other strains and media but at a reduced rate (Cowan I in TSB [2/7 populations]; 6850 in TSB [1/7]; JE2 in PA supernatant + TSB [1/7]). While screening for SCVs is challenging, as colony size is a variable trait and unstable SCVs can quickly revert to regular colony sizes (47), our results suggest that SCVs arose most commonly in Cowan I populations evolved in PA supernatant. We thus screened all evolved clones from this evolution condition and found that 5 out of the 25 Cowan I clones expressed an SCV phenotype. We classified three and two of these clones as dynamic/unstable and stable SCVs, respectively. Dynamic/unstable SCVs consistently formed two colony types (small and normal-sized colonies) upon repeated re-streaking. When comparing their growth trajectories in PA supernatant + TSB to the Cowan I ancestor, they had a shorter lag phase and a higher yield (Fig. 5A). The two stable SCVs had a longer lag phase and a lower yield than the normal-sized clones isolated from the same population. However, the death phase observed in the Cowan I ancestor was abrogated, suggesting that stable SCV formation increases survival under stressful conditions (Fig. 5B) such as exposure to PA supernatant.

**Fig 6:**
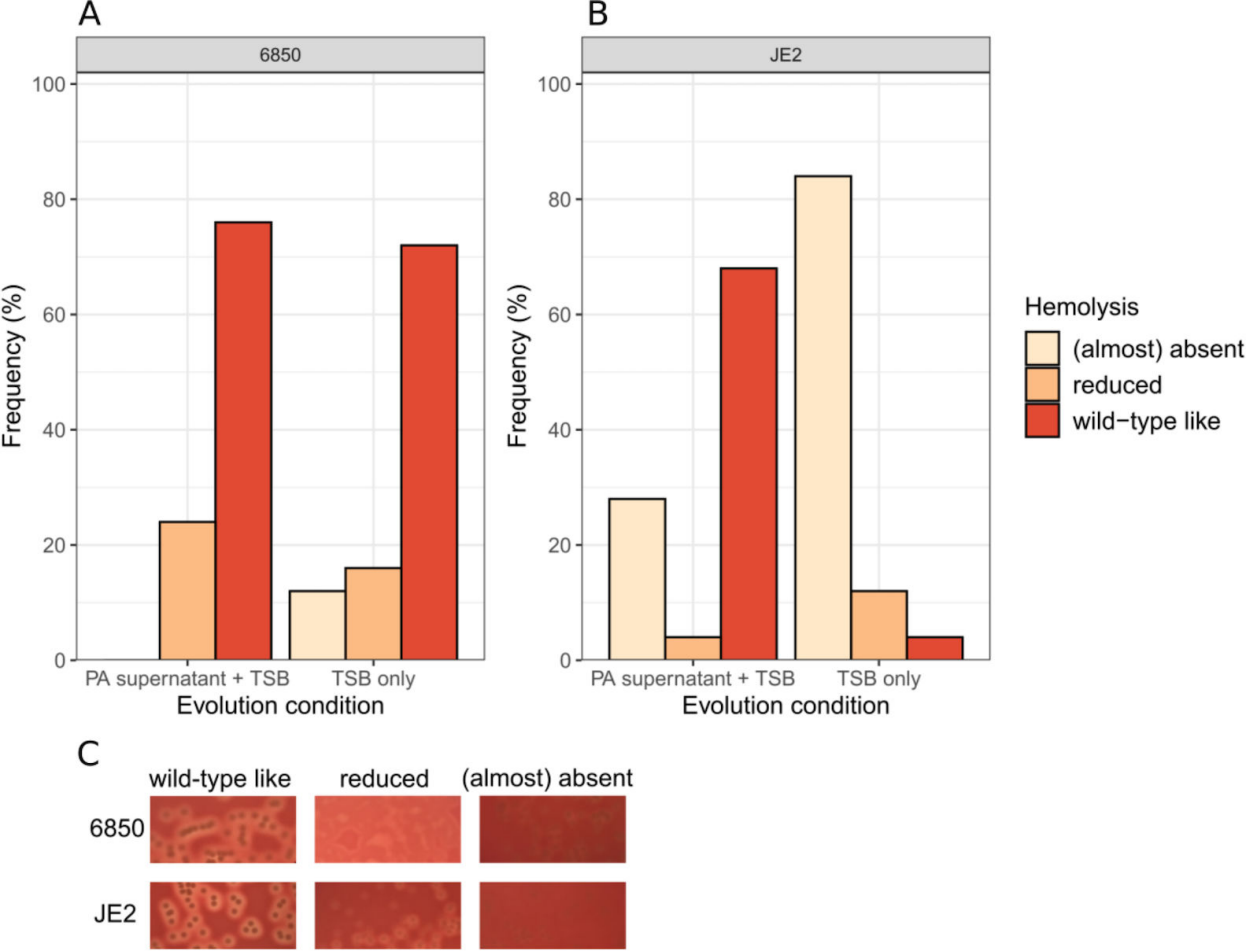
The PA supernatant selects for the maintenance of hemolysis in JE2. One hundred evolved clones from the SA strains 6850 and JE2 were plated on sheep blood agar plates to score their hemolysis levels according to three categories: wild-type like (ancestral), reduced, and (almost) absent. Note that the Cowan I ancestor is non-hemolytic and therefore not part of this assay. (**A**) Frequency of the three hemolysis categories for the evolved 6850 clones. There is no significant difference in the hemolysis profiles between clones evolved in 30% PA supernatant + 70% tryptic soy broth (TSB) vs 100% TSB (Fisher’s exact test: *P* = 0.2266) and most clones remained hemolytic. (**B**) Frequency of the three hemolysis categories for the evolved JE2 clones. There is a significant difference in the hemolysis profiles between clones evolved in PA supernatant + TSB vs in TSB only (Fisher’s exact test: *P* < 0.0001). The large majority of JE2 clones evolved in TSB only are no longer hemolytic. (**C**) Representative pictures of the three hemolysis categories for the SA strains 6850 and JE2.

Hemolysis (lysis of red blood cells) is an important virulence-related phenotype in SA, although the loss of hemolysis has repeatedly been observed in SA isolates from chronic infections (48 – 50). Here, we asked whether the presence of PA supernatant affects the evolution of this phenotype in SA, although hemolysis itself should play a minor role during experimental evolution in TSB. We focused on 6850 and JE2 (Cowan I is a non-hemolytic SA strain). Our population (Fig. S9) and clonal (Fig. 6) level analyses show that the majority of 6850 and JE2 clones and populations remained hemolytic after evolution in PA supernatant + TSB. In contrast, after evolution in 100% TSB, hemolytic activity only remained high in clones from 6850 (72% still showed ancestral activity, Fig. 6A), while 84% of the JE2 clones lost their hemolytic activity (Fig. 6B), a pattern that was also reflected at the population level (Fig. S9). These findings show that the presence of PA supernatant fosters the maintenance of hemolysis, and that the speed by which hemolysis can be lost depends on the SA strain genetic background.

### Mutational patterns of evolved clones are specific to evolution conditions

**Fig 7:**
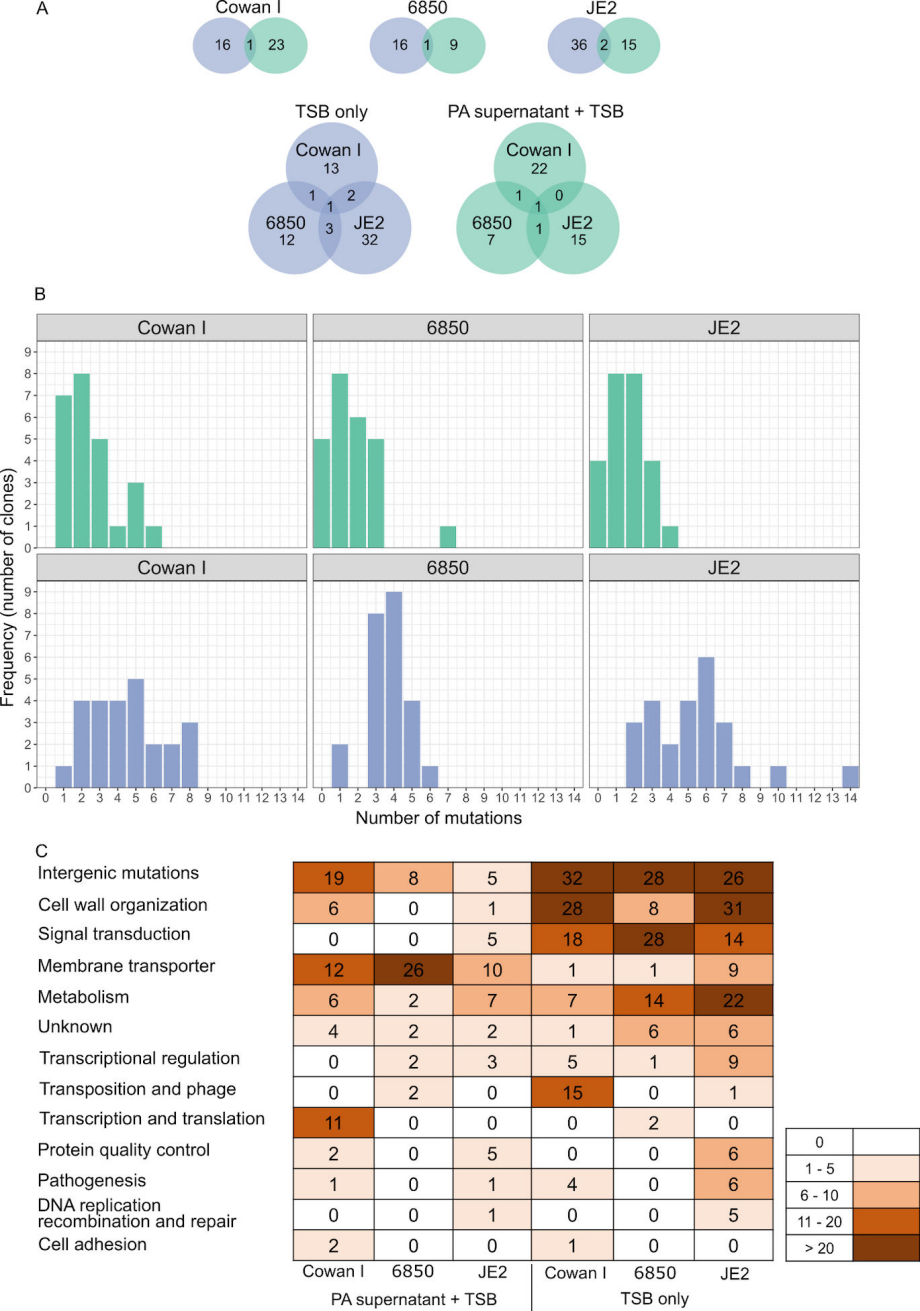
Genomic analysis of 150 evolved clones reveals strain and condition-specific mutational patterns. (**A**) Venn diagrams showing how many genes with non-synonymous mutations are unique or shared between evolution conditions (green: 30% PA supernatant + 70% tryptic soy broth (TSB); blue: 100% TSB) and the three SA ancestors (Cowan I, 6850, and JE2). (**B**) Number of mutations per clone (non-synonymous and intergenic mutations) split according to SA strain background and evolution condition. Clones evolved in the presence of PA supernatant had significantly fewer mutations than clones evolved in TSB alone. (**C**) Heatmap showing the total abundance of mutations in functional gene categories and intergenic regions (top row) across all sequenced clones, split by SA strain background and evolution condition. Note that we counted every mutation in every clone for this latter analysis.

To identify the genetic basis of evolutionary changes, we sequenced the genomes of the phenotypically characterized 150 evolved clones. Compared to the ancestors, we identified non-synonymous mutations in 119 different genes and mutations in 51 different intergenic regions. Among those, 68 and 109 genes or intergenic regions were mutated in clones that had evolved in PA supernatant + TSB and in 100% TSB, respectively. The large majority (96.6%) of the 119 genes with non-synonymous mutations occurred uniquely in one of the two media (either in PA supernatant + TSB or 100% TSB), and there was little overlap in mutational targets between strains (Fig. 7A). These results are in line with our phenotypic assays, highlighting that each SA strain followed a divergent evolutionary trajectory.

For all SA strains, the median number of mutations was lower for clones evolved in PA supernatant than in TSB alone (Cowan I: 2.0 vs 4.0; 6850: 1.0 vs 4.0; and JE2: 2.0 vs 5.0, Fig. 7B), indicating that fewer mutations were beneficial in the presence of PA supernatant than in TSB alone. The ratio of non-synonymous to synonymous SNPs (single nucleotide polymorphism; dN/dS) was higher than one for all strains and in both media (dN/dS for Cowan I: PA supernatant + TSB/100% TSB = 3.1/2.5; for 6850: 13.0/12.0; and for JE2: 3.6/4.0). These results show that positive selection and adaptive evolution occurred under both conditions.

To determine whether the mutational patterns differed between the two media and the three SA strains in terms of the functional classes, we sorted the mutated genes using aureowiki (51) and the primary literature. In clones evolved in PA supernatant, we found an enrichment of mutated genes belonging to the category “membrane transporter,” while for clones evolved in TSB alone, most mutated genes belonged to the categories “metabolism,” “cell wall organization,” and “signal transduction” (Fig. 7B).

### Parallel evolution in TSB, diverse nonparallel evolution in PA supernatant

We explored whether there is evidence for parallel evolution, which should manifest in similar mutational patterns surfacing across independently evolved populations and strains. For this purpose, we identified genes that mutated more than once in at least two populations/strains. Across both evolution conditions, we found 11 genes that fulfilled the criteria (Table S3). When focusing on clones evolved in TSB alone, there were three genes (*fmtA*, *gdpP*, and *walK*) that stood out in terms of both their frequency of mutations and distribution across SA strains. The teichoic acid D-Ala esterase *fmtA* mutated in five Cowan I populations, one 6850 population, and five JE2 populations (5/1/5). Mutations in the cyclic-di-AMP phosphodiesterase *gdpP* (0/4/5) and the cell wall histidine kinase *walK* (5/3/0) were similarly frequent. A striking pattern is that mutations in any of these three genes are only common in two out of the three SA strains in changing combinations. These results demonstrate that (i) there is high level of parallel evolution between populations of the same strain; (ii) there is a certain level of parallel evolution across strains but the combination of genes that are under selection varies; and (iii) mutations in *fmtA*, *gdpP,* and *walK* are likely involved in general adaptations to the TSB medium, as mutations in these genes are absent in clones evolved in PA supernatant.

We found little evidence for parallel evolution among populations evolved in PA supernatant. Only the gene *alsT* mutated in all SA strain backgrounds (Cowan I [two populations], 6850 [three], JE2 [one]), with *alsT* mutations occurring in a total of 22 clones. This suggests that mutations in *alsT* encoding a glutamine transporter (52) are generally advantageous in the presence of PA supernatant. The overall lack of parallel evolution indicates that SA populations and strains adapted to PA supernatant in a diverse manner.

### SA takes diverse mutational trajectories to evolve resistance to PA inhibitory molecules

**Fig 8:**
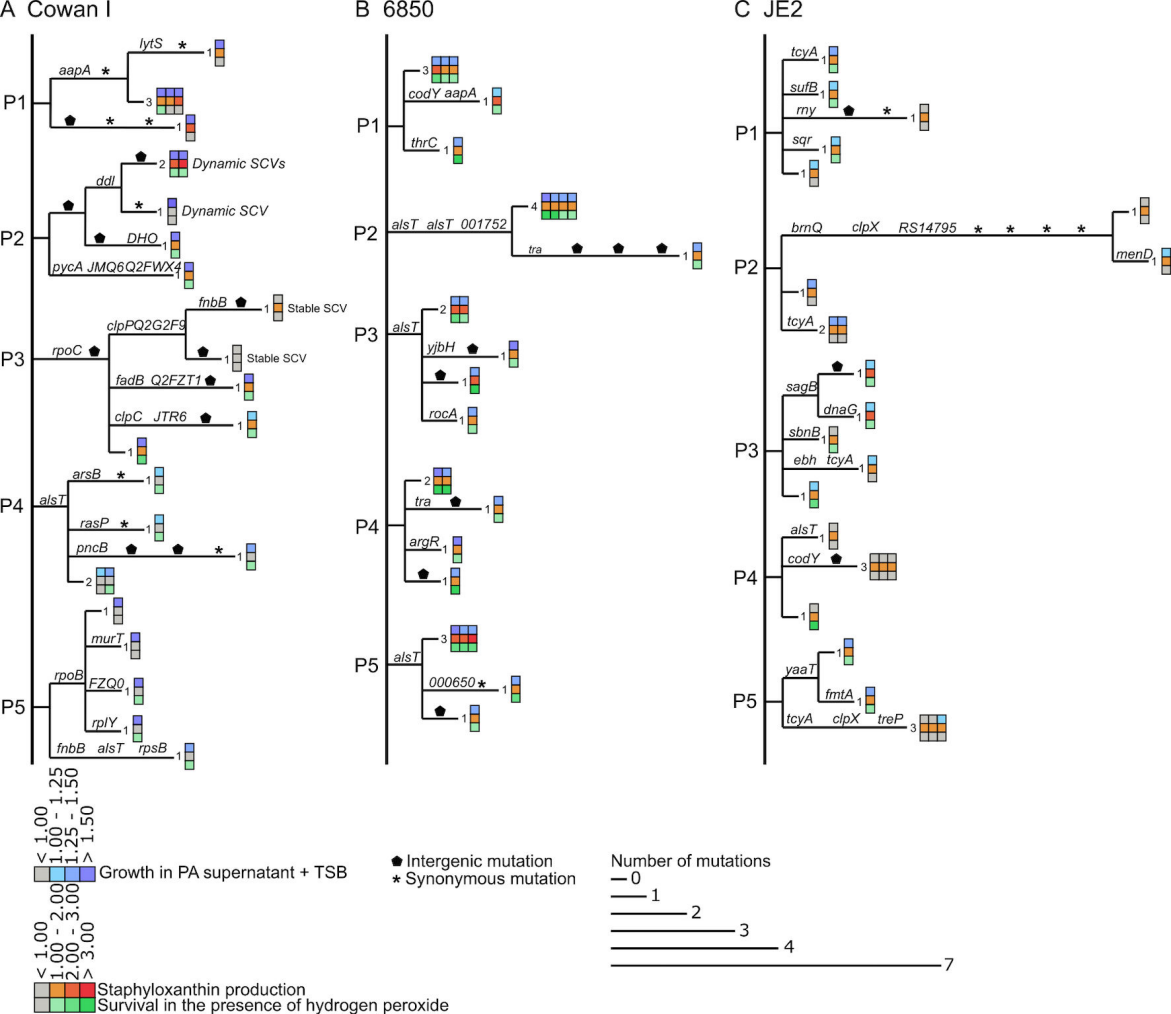
Cladograms showing within-population mutational patterns and their match to phenotypes of SA clones evolved in the presence of PA supernatant. Separate cladograms are shown for each of the five populations (P1–P5) featuring 25 clones of the three SA strains (**A**) Cowan I, (**B**) 6850, and (**C**) JE2. Branch length corresponds to the number of mutations per clone. Labels on branches indicate either non-synonymous mutations (gene name or abbreviated locus tag), intergenic mutations (pentagon), or synonymous mutations (asterisk). While all clones are from the final time point of the experiment, sequence of events can be inferred based on the presence/absence of mutations (synonymous, non-synonymous, and intergenic mutations) across clones from the same population. Heatmaps on the tip of the branches show growth in the presence of PA supernatant (top row), staphyloxanthin production (middle row), and survival in the presence of hydrogen peroxide (bottom row) for the respective evolved clones, all expressed relative to the ancestor.

We used the uncovered mutational patterns to construct evolutionary cladograms for each population evolved in PA supernatant. Additionally, we mapped three phenotypes (growth in PA supernatant + TSB, STX production, H_2_O_2_ survival) onto the cladograms to identify links between genotypic and phenotypic changes (Fig. 8).

For Cowan I (Fig. 8A), mutational patterns were highly diverse within and across populations and there was no apparent link to any of the phenotypes. For example, while 23 out of 25 clones showed resistance to PA supernatant in terms of growth, the underlying mutational patterns were extremely diverse. For 6850 (Fig. 8B), there is some evidence for parallel evolution via mutations in *alsT*, which became fixed in three different populations. All clones with *alsT* mutations showed higher growth, increased STX production, and H_2_O_2_ survival. For JE2 (Fig. 8C), the genotype-phenotype matching was complicated, too. Overall, JE2 showed the least pronounced changes in phenotypes with many of the clones not growing better than the ancestral wild type (Fig. S6). When focusing on the clones in populations P1, P3, and P5, which showed the most pronounced increase in growth, STX production and H_2_O_2_, we found completely different underlying mutational patterns in each population. For example, mutations in *tcyA* (population P1), *sagB* (P3), and *yaaT* (P5) all seemed to be associated with the altered phenotypes. The *tcyA* gene, encoding a membrane transporter, stood out from the other genes as it was repeatedly mutated in seven clones from four different JE2 populations, suggesting that it could play a prominent role in SA resistance evolution.

## DISCUSSION

PA and SA are among the most troublesome pathogens in polymicrobial infections, such as wound and CF lung infections. That is the reason why there is high interest in understanding how species interactions shape disease outcome (3, 9, 53, 54). A large body of literature has examined PA-SA interactions at the molecular level, and mostly described PA as the dominant species suppressing the growth of SA by producing a variety of inhibitory molecules (8). However, polymicrobial infections containing PA and SA are often chronic so that the two species may adapt to one another, and SA may become resistant to PA inhibitory molecules (55, 56). Here, we show that the molecules PQS and HQNO secreted by PA are involved in the inhibition of three different SA strains (Cowan I, 6850, and JE2). During experimental evolution, we found that all SA strains rapidly evolved resistance to the PA-induced growth inhibition. We observed that (i) resistance was associated with the upregulation of traits involved in the protection from reactive oxygen, including STX production and the formation of SCV, (ii) adaptive patterns were SA strain-specific, and (iii) mutations in genes encoding membrane transporters were the most frequent target of evolution. Thus, our results suggest that resistance is achieved by a combination of decreased influx of harmful PA compounds and direct protective measures against oxidative stress. While these evolutionary changes might be specific to our experimental setup, they concern the expression of virulence traits (STX and SCV) and membrane transporters and could thus affect virulence and antibiotic susceptibility phenotypes if they arise in infections (57 – 59).

We found that the *Pseudomonas* quinolone signaling pathway, controlling the production of PQS and HQNO, was involved in growth inhibition. PQS does not only serve as a quorum-sensing signaling molecule but also functions as an iron trap (60) and triggers the formation of reactive oxygen species (38 – 41). The iron-chelating activity of PQS reduces the bioavailability of iron in the environment, which was shown to inhibit a variety of gram-negative and gram-positive bacteria (35, 61). While the induction of iron limitation could have contributed to the observed growth inhibition in our experiments, our data rather suggest that inhibition was mainly due to the ability of PQS to trigger the formation of reactive oxygen species (Fig. 3; Fig. S6). HQNO is known to have anti-SA properties by inhibiting the respiratory chain (26).

We found that the extent to which SA strains were inhibited and the strength of resistance evolution toward the PA supernatant varied across SA strains (Fig. 3). While the Cowan I ancestor was substantially inhibited, it showed the largest improvement in growth after evolution in the PA supernatant. Similarly, 6850 was initially greatly inhibited by the PA supernatant, and all replicate populations showed resistance evolution, reflected by a significantly improved growth. JE2 was initially the least inhibited strain, and while overall, we found significantly improved growth after evolution, not all replicate populations followed this trend. This differential response matches evolutionary predictions that the most severely affected SA strains face the strongest selection pressure and consequently show the strongest response in terms of resistance evolution. Moreover, JE2 is the most virulent and most competitive SA strain (relative to PA) in our panel (21, 62), leaving less room for further improvements compared to the less virulent and less competitive Cowan I and 6850 strains.

At the genetic level, we found evidence for positive selection but few signs for parallel evolution across strains and populations upon exposure to the PA supernatant. This indicates that mutations in many different genes were under selection and possibly contributed to resistance evolution toward PA inhibitory molecules. The fact that mutations in a broad spectrum of genes can promote resistance highlights the enormous evolvability of SA (63). It also shows that phenotypic and genetic diversification arises quickly in SA populations (Fig. 8). The co-existence of genetic variants is often overlooked in a clinical context but is increasingly recognized as both prevalent and important for disease outcomes (64, 65). In contrast, parallel evolution occurred among clones that evolved in the TSB medium alone. We identified several genes (Table S3) that were frequently mutated in independent populations and across strains, indicating that medium adaptation is more predictable than adaptation to a competitor.

Our sequencing results revealed that many mutations occurred in membrane transport genes when SA strains were exposed to PA supernatant (Fig. 7C). Given that mutations arising during relatively short experimental evolution experiments are often associated with reduced or loss of function, we discuss two specific examples in this context. The gene encoding the glutamine transporter AlsT was mutated in 22 clones from six populations across all three SA strains. While AlsT inactivation and loss of function reduce the susceptibility of SA to the toxic glutamine analog γ-L-glutamyl hydrazide (52), it also increases intracellular c-di-AMP levels, which allows bacteria to better resist stressful conditions (66). In 6850, there were five clones that had only *alsT* mutated, and all showed strongly improved growth in the PA supernatant (Fig. 8). These results support the view that AlsT modifications might indeed reduce the uptake of PA inhibitory molecules and thereby improve the ability of SA to withstand the stress imposed by PA. Another example involves mutations in the gene *tcyA*, which encodes a part of the cystine transporter TcyABC. While mutations in this gene were unique to JE2, they surfaced in seven clones in four out of the five JE2 populations. Three out of these clones had no other mutations apart from those in *tcyA*, suggesting that alterations in the TcyABC transporter are responsible for the observed growth increase and resistance to the inhibitory molecules of PA (Fig. 8). Previous work showed that deletion of *tcyA* leads to resistance against the toxic cystine analog selenocystine in SA (67). Our results now suggest that a reduced or loss of function of TcyA could also protect SA from other toxic compounds such as PQS and HQNO. Altogether, mutational changes in AlsT and TcyABC transporters could have similar effects as mutations in porin channels that are often observed in the context of antibiotic resistance (68): they offer immediate protection against toxic compounds without the need for any priming to occur and tolerance to build up.

In summary, our work shows that SA rapidly and specifically adapts to PA inhibitory molecules. Several virulence traits were upregulated (STX, SCV) or selectively maintained (hemolysis) in response to PA supernatant exposure, and our genomic analysis revealed that mutations in membrane transport genes are associated with resistance evolution in SA. While these evolutionary patterns were observed in a laboratory experiment, they indicate that if similar adaptations occur in infections, there could be several complications. For example, SA virulence could be increased, and baseline levels of antibiotic resistance might arise due to loss-of-function mutations in importers. In a next step, it would be important to study co-evolution and its consequences for reciprocal adaptation in both species, as co-evolution is likely to occur in polymicrobial infections (54). In a broader sense, the fact that evolutionary trajectories were strongly SA strain-specific suggests that we ultimately need to understand polymicrobial infection dynamics and its consequences at the individual patient level to improve treatment options.

## MATERIALS AND METHODS

### Bacterial strains, media, and growth conditions

For all main experiments, we used PA strain PAO1 (ATCC 15692) and the three genetically different SA strains Cowan I, 6850, and JE2 [Table S1, see also references (21, 69) for further information and strains and species interaction]. We further used a set of two different PA mutants. First, we used gene deletion mutants (PAO1*ΔpvdDpchEF*, *ΔlecA*, *ΔlasR*, *ΔrhlR*, *ΔrhlI*, *ΔlrhlA*) that directly originated from the above PAO1 wild type. Second, we used transposon mutants (MPAO1*pqsA::Tn*, *pqsH::Tn*, *pqsL::Tn*) from the Manoil mutant library and the corresponding wild-type MPAO1 (33). These mutants were kindly provide to us by the Häussler lab (39).

All growth experiments and the experimental evolution were performed in TSB (Becton Dickinson, Heidelberg, Germany) medium. Overnight cultures (prior to experimental evolution) were grown in 10 mL TSB in 50 mL falcon tubes at 37°C and 220 rpm with aeration. The phenotypic assays after experimental evolution involved large sample sizes including many evolved clones, populations, and the respective ancestors. For this reason, we grew overnight in 24-well plates filled with 1.5 mL TSB per well at 37°C and 170 rpm.

### Supernatant assays

To test whether the supernatant of PA inhibits SA (and vice versa), we performed supernatant growth assays as follows. We first generated cell-free supernatants by growing PA and SA donor strains overnight in TSB. After centrifugation of the cultures to pellet cells, we sterile-filtered the supernatant using 0.2 µM pore size filters (Whatman, Fisher Scientific, Reinach, Switzerland) and stored aliquots of the supernatant at −20°C. For the supernatant assay, we grew PA and SA receiver strains overnight, washed bacterial cell pellets with 10 mL 0.8% NaCl, and adjusted OD_600_ to obtain comparable cell numbers per milliliter for all strains. To achieve this, we adjusted OD_600_ of PA to 1.0, for SA strains JE2 and 6850 to 0.4, and for Cowan I to 0.8. Subsequently, we diluted the PA inoculum 1:10^5^ and the SA inoculums 1:10^4^ to start our experiments. PA was diluted more because it grows faster compared to SA. The diluted inoculums were prepared in the following three media conditions: (1) 100% TSB (as growth control) (2); 30% NaCl solution (0.8%) + 70% TSB (control to mimic reduced nutrient availability) (3); 30% PA or SA supernatant + 70% TSB (condition of interest). We used 30% supernatant based on our previous experience showing that this ratio represents a good balance between providing enough fresh medium so that bacteria show decent growth and adding a high enough concentration of secreted compounds so that bacteria are affected (70).

Supernatant assays were performed in 96-well plates with 200 µL per well. We incubated plates statically at 37°C in a plate reader (Tecan Infinite M Nano, Tecan, Männedorf, Switzerland) and measured growth of strains by recording OD_600_ every 30 minutes for 48 hours, with 30 seconds shaking events prior to recordings. Effect of PA supernatant on growth of SA strains was assessed in seven independent experiments, and effect of SA supernatants on PA growth was assessed in one experiment per SA supernatant. In every assay, we used four replicates per strain and growth condition.

### SA growth in the presence of PQS

To test whether PQS (heptyl-3-hydroxy-4(1H)-quinolone, Sigma-Aldrich, Buchs, Switzerland) is involved in the growth inhibition of SA, we exposed cultures of all three SA strains to final concentrations of 20 µM, 50 µM, or 80 µM PQS (10 mM stock in DMSO), supplemented to the TSB growth medium, and compared their growth relative to the growth in TSB supplemented with DMSO alone (same volume of DMSO as for the 80 µM PQS treatment). SA strain preparation and growth measurements (OD_600_) in 200 µL medium volume in 96-well plates followed the exact same protocol as for the supernatant assays using a plate reader.

Following evolution, we repeated the above experiment for 150 evolved clones (see below for the selection procedure) to test whether SA strains have evolved resistance to the growth inhibition induced by PQS. For this experiment, we exposed each individual clone (grown overnight in TSB) to either 50 µM PQS or DMSO (as a control) in triplicates. We followed the same protocol as above, except that we incubated the plates in a shaken incubator at 170 rpm and measured growth at the final time point after 48 hours.

### Experimental evolution of SA in the presence or absence of PA supernatant

To elucidate whether and how SA strains respond to the presence of PA supernatant over time, we performed an experimental evolution experiment, in which we exposed the three SA strains Cowan I, 6850, and JE2 for 30 days to either 30% PA supernatant + 70% TSB (experimental treatment) or 100% TSB (control treatment). Experimental evolution started from clonal populations. We had seven independent populations per strain and condition, resulting in a total of 42 evolving populations (three SA strains × two conditions × seven replicates). Prior to experimental evolution, overnight cultures were grown in TSB, washed, and OD-adjusted as described above. The starting inoculum for all strains and populations was 2 × 10^4^ cells. Cells were distributed into the respective medium on 96-well plates. We had each SA strain on a separate plate to avoid contamination. Plates were incubated statically at 37°C. Every 48 hours, we diluted and transferred the evolving cultures 1:10,000 into fresh medium and recorded OD_600_ as a proxy for growth prior to each transfer using a plate reader (Tecan Infinite M Nano). This dilution factor was chosen to bring cultures back to their initial cell density without the risk of inducing strong bottlenecks. To the part of the culture that was not transferred, we added equal volumes of a sterile 85% glycerol solution directly into the wells of the 96-well plate and froze them at −80°C as a backup. Overall, the experiment ran for 30 days (15 transfers). Based on a pre-experiment, we estimated the mean number of doublings (± standard deviation) for the three SA ancestors in the two growth conditions during a 48 hours growth cycle as follows: for Cowan I: 14.5 ± 0.4 (in TSB)/9.2 ± 0.7 (in PA supernatant + TSB); for 6850: 14.3 ± 0.8/10.6 ±1.7; for JE2: 14.6 ± 0.5/13.5 ± 0.6. Thus, our experimental evolution involved at least 140–220 bacterial generations (assuming no evolutionary improvements in growth). Following experimental evolution, we plated all evolved populations on 1.2% TSB agar and randomly picked five clones per population for further characterization. All selected clones were first cultured overnight in TSB and then frozen at −80°C by mixing 50% of culture with 50% of a sterile 85% glycerol solution.

### Growth of evolved populations and clones

We tested whether the 42 evolved populations and the selected 210 clones showed improved growth performance relative to the ancestors in the two conditions of our experimental evolution (30% PA supernatant + 70% TSB and 100% TSB). We exposed all populations and clones to the condition they had evolved in but also to the alternate condition they had not evolved in. For this growth screening, we followed the exact same protocol as the one described above for the supernatant assay.

### STX quantification

For the subsequent phenotypic and genotypic assays, we used a total of 150 clones from 30 populations (five clones per population). Sample size was reduced to meet the sequencing contingent of this project. In a first assay, we tested whether evolved clones changed STX production compared to the respective ancestor and quantified STX based on a previously published protocol (43). Briefly, we first collected 1 mL from each culture of all evolved clones and ancestors (grown overnight in TSB) and resuspended washed pellets in 400 µL methanol. We incubated the samples for 10 minutes at 55°C, pelleted cells by centrifugation, and collected the supernatant containing the STX. Absorbance of the crude extract was measured in duplicates at λ = 465 nm using a plate reader (Tecan Infinite M200 Pro). We blank-corrected STX values and expressed them first, relative to the blank-corrected OD_600_ of the respective clone and second, relative to the respective STX value of the ancestor.

### H_2_O_2_ survival assay

To determine whether evolved clones are better at surviving under oxidative stress conditions than the respective ancestors, we performed a H_2_O_2_ survival assay using a previously published protocol (71). In short, we subjected cultures of all evolved clones and ancestors (grown overnight in TSB) to either 1.5% H_2_O_2_ or 0.8% NaCl (as control) for 1 hour at 37°C. Subsequently, we plated appropriate dilutions on 1.2% TSB agar, incubated plates overnight at 37°C, and enumerated colony forming units (CFUs) on the next day. We then calculated the percentage survival for each clone as the CFUs from the H_2_O_2_ treatment divided by the CFUs from the control treatment multiplied by 100. Finally, we expressed survival relative to the respective ancestor.

### SCV detection and quantification

To determine whether evolved clones express SCV phenotypes, we plated cultures of all evolved clones on 1.2% TSB agar, incubated plates overnight at 37°C, and then classified the colonies with an area smaller than 20% of the ancestral colony size as SCV. To estimate the prevalence of SCVs at the population level, we also serially diluted and plated aliquots of every evolved population on 1.2% TSB agar and assessed the percentage of SCVs per population after incubation of plates overnight at 37°C.

### Hemolysis assay

The ancestral strains of 6850 and JE2 are known for their ability to lyse red blood cells (hemolysis), whereas Cowan I is unable to do so. To determine whether evolved clones of 6850 and JE2 show different hemolysis patterns compared to their ancestors, we plated cultures of all evolved clones and ancestors (grown overnight in TSB) on COS plates (columbia agar + 5% sheep blood, Biomérieux, Petit-Lancy, Switzerland). COS plates were incubated overnight at 37°C. On the next day, we qualitatively assessed the hemolysis pattern of each clone according to three discrete categories: ancestral-like hemolysis (wild-type like), reduced hemolysis, and (almost) absent hemolysis (examples depicted in Fig. 6C). In addition, we also plated all evolved populations on COS plates to assess the frequency of the three hemolysis phenotypes within populations.

### Genome sequencing

To identify genetic changes in evolved clones relative to their ancestors, we extracted the genomic DNA of all 150 phenotypically characterized clones and the three ancestral SA strains. We used the Maxwell RSC Cultured Cells DNA Kit (Promega, Dübendorf, Switzerland) together with the Maxwell RSC 48 instrument (Promega) following the manufacturer’s protocol. To lyse the gram-positive SA cells, we added lysostaphin (L7386, Sigma-Aldrich) to the samples (final concentration of 25 µg per 400 µL sample). DNA concentrations were quantified using the QuantiFluor dsDNA Sample Kit (Promega). Library preparation was performed with 100 ng of DNA input using the TruSeq DNA Nano Kit (Illumina, San Diego, USA) according to the manufacturers’ instructions. The libraries were quantified using the Tapestation (Agilent Technologies, Santa Clara, USA) and qPCR (Roche, Rotkreuz, Switzerland) and equimolarly pooled. Finally, the libraries were sequenced 150 bp paired-end on the NovaSeq 6000 system (Illumina, San Diego, USA). The samples had an estimated coverage of 100× for the evolved clones and 200× for the three SA ancestors.

The quality of the raw sequencing data were assessed using FastQC (version 0.11.9) (https://www.bioinformatics.babraham.ac.uk/projects/fastqc/) and a contamination check was performed using FastQ Screen (version 0.14.1). Subsequently, the raw reads were pre-processed using Trimmomatic (72) with the following settings: trimmString=”ILLUMINACLIP: adapters.fa:1:30:10 LEADING:15 TRAILING:15 SLIDINGWINDOW:5:30 AVGQUAL:32 MINLEN:80”. All the reads that passed the trimming and quality filtering steps were analyzed using Snippy (https://github.com/tseemann/snippy) (version 4.5.2) with default parameters. For the ancestral strains 6850 and JE2, the reads were aligned to the published reference genomes (GenBank accession Nr. CP006706.1 and NZ_CP020619.1, respectively). Variants that were present in the ancestor compared to the published reference genome were removed and only those variants that occurred during experimental evolution were kept. Finally, we used SnpEff version 4.3t (built 2017–11-24 10:18) (https://pcingola.github.io/SnpEff/) to predict variant effects. For Cowan I, no suitable reference genome was available, and we thus performed a *de novo* assembly with SPAdes (version 3.12.0) and annotation with Prodigal (version 2.6.3) using the reads from our ancestor, and then directly called the variants as in the other two strains for the evolved clones relative to the *de novo* assembled ancestral genome.

### Statistical analysis

All statistical analyses were performed with R Studio version 3.6.3. We used spline fits to analyze OD_600_ growth trajectories of SA strains over 48 hours. Non-parametric curve fits performed best because OD-values did often not reach a stable stationary phase after 48 hours (Fig. 1A and B). For growth curve analyses across shorter time intervals (Fig. 1C), we fitted logistic models to the growth curves as these models performed well. For both type of analyses (spline and logistic analyses), we calculated the area under the curve (growth integral) and the lag phase as the two most relevant growth parameters for our analysis.

We used general linear models wherever possible and consulted Q-Q plots and the Shapiro-Wilk test to examine whether residuals were normally distributed. The basic linear model that we used was an ANOVA in which we put the response variable in relation to the manipulated factors, which are (i) the SA strain background (Cowan I, 6850, and JE2), (ii) the experimental conditions (presence/absence of PA supernatant or PQS concentration), and (iii) the interaction between the two. We used variants of this model for statistical analysis of all growth data, STX production, and survival in the presence of H_2_O_2_ as response variables. Non-significant interactions were removed from the models. If data were not normally distributed (as for the survival data), we log_10_(× + 1)-transformed all values for statistical analysis and scaled them back to the respective ancestor for plotting. For all statistical tests, we provide the test value, the degrees of freedom, and the *P*-value. All tests are two-sided. The false discovery rate method was used to correct *P*-values whenever necessary. For frequency comparisons between hemolysis categories for 6850 and JE2 clones evolved in the two media, we performed Fisher’s exact tests. The PCA was performed on clonal phenotypes using the *vegan* package in R. We tested for inference using PERMANOVA.

## Data Availability

All source data associated with this study is available from the figshare repository, doi:10.6084/m9.figshare.23578086. The genome sequencing data is deposited in the European Nucleotide Archive (https://www.ebi.ac.uk/ena/) under accession PRJEB63527.

## SUPPLEMENTAL MATERIAL

Supplemental Figures and Tables mbio.03153-22-s0001.docx Nine supplemental figures and three supplemental tables.

ASM does not own the copyrights to Supplemental Material that may be linked to, or accessed through, an article. The authors have granted ASM a non-exclusive, world-wide license to publish the Supplemental Material files. Please contact the corresponding author directly for reuse.
